# A molecular switch in immunodominant HIV-1-specific CD8 T-cell epitopes shapes differential HLA-restricted escape

**DOI:** 10.1186/s12977-015-0149-5

**Published:** 2015-02-20

**Authors:** Henrik N Kløverpris, David K Cole, Anna Fuller, Jonathan Carlson, Konrad Beck, Andrea J Schauenburg, Pierre J Rizkallah, Søren Buus, Andrew K Sewell, Philip Goulder

**Affiliations:** KwaZulu- Natal Research Institute for Tuberculosis and HIV, K-RITH, Nelson R Mandela School of Medicine, University of KwaZulu-Natal, Durban, South Africa; Cardiff University School of Medicine, Heath Park, Cardiff, UK; Microsoft Research, eScience Group, Los Angeles, CA 90024 USA; Cardiff University School of Dentistry, Heath Park, Cardiff, UK; Department of International Health, Immunology and Microbiology, University of Copenhagen, Copenhagen N, 2200 Denmark; Department of Paediatrics, University of Oxford, Peter Medawar Building, Oxford, OX1 3SY UK

**Keywords:** HIV-1, CD8^+^ T-Cells, Viral escape, pHLA structure

## Abstract

**Background:**

Presentation of identical HIV-1 peptides by closely related Human Leukocyte Antigen class I (HLAI) molecules can select distinct patterns of escape mutation that have a significant impact on viral fitness and disease progression. The molecular mechanisms by which HLAI micropolymorphisms can induce differential HIV-1 escape patterns within identical peptide epitopes remain unknown.

**Results:**

Here, we undertook genetic and structural analyses of two immunodominant HIV-1 peptides, Gag_180–188_ (TPQDLNTML, TL9-p24) and Nef_71–79_ (RPQVPLRPM, RM9-Nef) that are among the most highly targeted epitopes in the global HIV-1 epidemic. We show that single polymorphisms between different alleles of the HLA-B7 superfamily can induce a conformational switch in peptide conformation that is associated with differential HLAI-specific escape mutation and immune control. A dominant R71K mutation in the Nef71-79 occurred in those with HLA-B*07:02 but not B*42:01/02 or B*81:01. No structural difference in the HLA-epitope complexes was detected to explain this observation.

**Conclusions:**

These data suggest that identical peptides presented through very similar HLAI landscapes are recognized as distinct epitopes and provide a novel structural mechanism for previously observed differential HIV-1 escape and disease progression.

**Electronic supplementary material:**

The online version of this article (doi:10.1186/s12977-015-0149-5) contains supplementary material, which is available to authorized users.

## Background

The human leukocyte antigen (HLA) locus on chromosome 6 is the most polymorphic region of the human genome. The extreme diversity of HLA class I (HLAI) loci allows optimal binding of peptides derived from the vast array of environmental pathogens [1]. The HLAI residues that are polymorphic are mainly those forming the peptide-binding groove, which contains six binding pockets (A to F) that define the size and chemical characteristics of the specific peptide repertoire that can be accommodated by each HLAI molecule. Interaction between peptide and HLAI is usually governed by the compatibility of residues at the N- and C-terminus of the peptide (peptide anchor residues) within the highly polymorphic binding pockets. Cognate T-cell receptors (TCRs) expressed on CD8^+^ T-cells detect pathogen-derived peptides presented by HLAI molecules on the surface of infected cells [2].

Minor differences between the HLAI molecules expressed (‘HLAI micropolymorphisms’), even of a single amino acid, can have a profound impact on both T-cell immunity and disease outcome during a range of infections, including HIV-1 [3-8]. This disparity has been partly explained by the selection of escape mutations that have different consequences for viral fitness [8-10]. For example, the dominant HIV-1-specific epitope restricted by two closely-related HLAI molecules within the B7 superfamily, HLA-B*42:01 and HLA-B*81:01, is the same peptide, Gag_180–188_ TPQDLNTML (TL9-p24) [11,12]. The escape mutation selected in each case differs, with variation most commonly arising at Gag-182 (position-3 in the epitope) in HLA-B*42:01-positive subjects, and at Gag-186 (position-7) in HLA-B*81:01-positive subjects. *In vitro* studies indicate that the escape mutants selected at Gag-186 have a dramatic negative impact on viral replication capacity, in contrast to the minimal effect of mutation at Gag-182 [13]. HLA-B*81:01, together with HLA-B*57 and HLA-B*58:01, is one of the group of HLAI molecules most strongly associated with immune control of HIV-1 [5,14,15] and the viral replicative capacity in HLA-B*81:01^+^ subjects has been reported to be lower than in subjects expressing any other allele [13]. The mutation principally responsible for this protective effect is the above-mentioned HLA-B*81:01-driven T186S variant that fails to yield replicating virus stocks in studies of C clade virus [13]. This is in line with the lower viral loads and higher CD4 counts observed in HLA-B*81:01 positive subjects in studies of exclusively C-clade infection [9,14,16]. Notably, in a study of cohorts in Zambia, Rwanda and Kenya involving several clades of virus, including clade A which carries a different consensus residue at Gag-186, stable CD4 counts but not lower viraemia was observed in association with HLA-B*81:01 [15].

In this study we focus on two HIV-1-specific epitopes and their presentation by 4 different members of the HLA-B7 superfamily, HLA-B*07:02, HLA-B*42:01, HLA-B*42:02 and HLA-B*81:01. These alleles are highly prevalent in Sub-Saharan Africa, the region worst afflicted by the HIV-1 pandemic, one or more being expressed in 35-40% of people comprising these populations [14]. The identical TL9-p24 and Nef_71–79,_ RPQVPLRPM (RM9-Nef) epitopes are presented by these alleles, and represent two of the dominant HIV-1-specific responses in Southern African study cohorts [14], being recognised by approximately 70% and 40%, respectively, of subjects expressing HLA B7-supertype alleles, and targeted by approximately 25% and 20%, respectively of *all* subjects in Southern African study cohorts, irrespective of HLAI type [14,17].

In the case of both epitopes, TL9-p24 and RM9-Nef, distinct patterns of escape mutations are induced by different HLAI molecules within the B7 superfamily [17]. We here have undertaken a genetic and structural approach to better understand the molecular mechanisms by which HLAI micropolymorphisms can influence the precise nature of T-cell escape and immune control through identical HIV-1 epitopes. These data demonstrate that even a single HLAI polymorphism can switch the conformation of a peptide in the HLAI binding groove, substantially altering both the peptide residues positioned to contact incoming TCRs, and consequently of the impact of variation at different residues within the same epitope.

## Results

### HLAI micropolymorphisms result in distinct targeting frequencies and selection pressure through two HIV-1 epitopes

**Figure 1:**
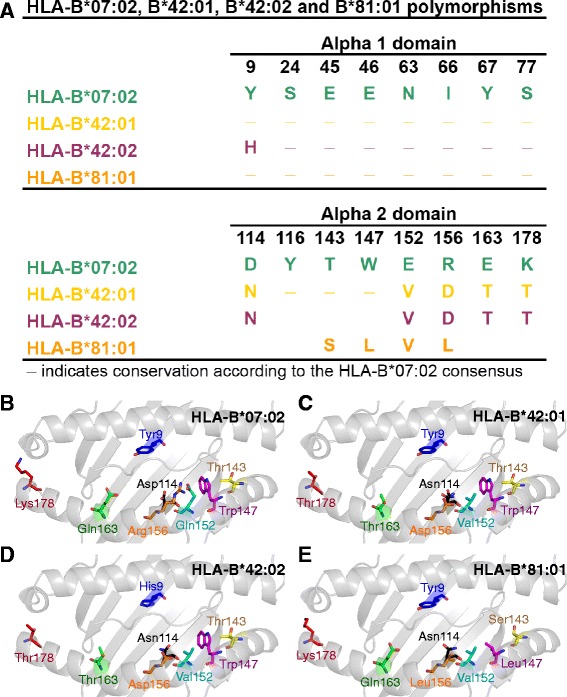
Micropolymorphisms within HLA-B*07:02, HLA-B*42:01, HLA-B*42:02 and HLA-B*81:01 molecules. (A) HLAI residue polymorphisms within α1 domain (top) and α2 domain (bottom) aligned to HLA-B*07:02. **(B-E)** The HLAI peptide binding groove shown as grey cartoon with each position in each HLAI coloured individually and shown as sticks for **(B)** HLA-B*07:02, **(C)** HLA-B*42:01, **(D)** HLA-B*42:02 and **(E)** HLA-B*81:01, at atomic resolution.

We studied four members of the HLA-B7 superfamily, HLA-B*07:02, HLA-B*42:01, HLA-B*42:02 and HLA-B*81:01. These closely-related HLAI molecules restrict both distinct and identical HIV-1 epitopes [4,7,18]. The differences in sequence between HLA-B*07:02, B*42:01, B*42:02 and B*81:01 are small (Figure 1A). Atomic resolution of these HLA-B molecules (crystallographic statistics in Additional file 1: Tables S1 and S2) demonstrate that all of the polymorphic positions are part of the HLAI peptide binding pockets (Figure 1B-E) and therefore potentially affect peptide presentation to CD8^+^ T cells.

**Figure 2:**
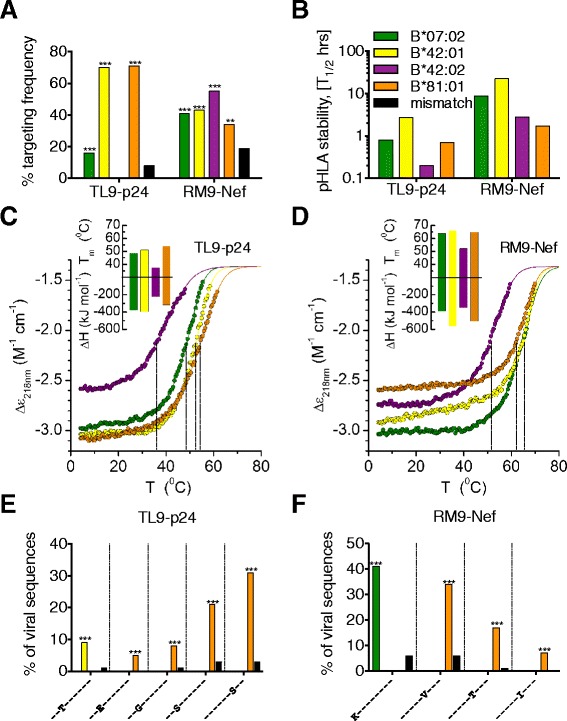
TL9-p24 and RM9-Nef immunodominance, pHLAI stability and differential selection pressure for HLA-B*07:02, HLA-B*42:01, HLA-B*42:02 and HLA-B*81:01. (A) Unbiased screening of n = 1,009 individuals with OLP-25 (GA*TPQDLNTML*NTVGGH) containing TL9-p24 and OLP-76 (EVGFPV*RPQVPLRPM*TFK) containing RM9-Nef of which n = 76 where HLA-B*07:02^+^, n = 154 where HLA-B*42:01^+^, n = 22 where HLA-B*42:02^+^ and n = 96 where HLA-B*81:01^+^. Individuals expressing HLA-B*39:10 (n = 30) were excluded as they also target TL9-24 and RM9-Nef. **(B)** TL9-p24 (TPQDLNTML) and RM9-Nef (RPQVPLRPM) pHLAI stability (binding half-life, h) against indicated HLA-B molecules. **(C, D)** CD thermal denaturation curves recorded at 218 nm are shown for selected pHLAI samples. Dots represent measured values fitted assuming a 2-state trimer-to-monomer transition (solid lines) as described in Methods. Insets show bar graphs of the thermal stability with respect to melting temperature (upper) and van’t Hoff’s enthalpy of unfolding (lower panel) using the same color code as in B. **(E, F)** Intraepitope HIV-1 polymorphisms for **(E)** TL9-p24 shown on the X-axis for Q182X as peptide position 3 and T186S as position 7 and **(F)** for RM9-Nef R71K as for peptide position 1 and L76X as position 6 and expressed as the percentage of total viral sequences for n = 1,327 HIV-1 infected individuals of which n = 189 expressed HLA-B*07:02, n = 436 expressed HLA-B*42:01, n = 56 expressed HLA-B*42:02 and n = 213 expressed HLA-B*81:01. Only Q < 0.05 is shown with ***indicating P < 0.0002 and **P < 0.002 compared to HLAI mismatched individuals.

To better understand the mechanisms underlying the observed differential HIV-1 selection pressure imposed on HIV-1 by distinct HLAI molecules within the B7 superfamily, we focused on two epitopes, TL9-p24 and RM9-Nef, that dominate the HIV-1-specific CD8 T-cell response in the Southern African HIV-1 epidemic [14,17]. These two epitopes were presented by all four of these HLAI molecules, other than TL9-p24 which was presented by all except HLA-B*42:02 (Figure 2A). TL9-p24, dominantly targeted through HLA-B*42:01 and HLA-B*81:01 and subdominantly through HLA-B*07:02, had a peptide-HLAI (pHLAI) stability of >0.7 h and T_m_ = 49-55°C, whereas the lack of TL9-p24 targeting via HLA-B*42:02 was associated with low pHLAI stability (<0.2 h, T_m_ = 37°C) [7]. RM9-Nef, however, was presented by all 4 different HLAIs, with pHLAI stabilities ranging from 1.7 h to 22.4 h (T_m_ = 52-66°C) (Figure 2B-D).

Sequence analysis of >1,200 HIV-1 C-clade infected individuals confirmed striking differences in HLAI-driven selection pressure within both epitopes (Figure 2E) and (Table 1). Of note, selection for the above-mentioned T186S at position 7 of the TL9-p24 epitope (31%, P = 2×10^−24^) was only significant in HLA-B*81:01-positive individuals. Distinct patterns of selection were also observed for RM9-Nef, even though this epitope was similarly targeted irrespective of the B7 superfamily HLA-B allele expressed (34%-54%). HLA-B*07:02 expressing individuals exhibited strong selection at P1 in the epitope (R71K: 41%, P = 7×10^−5^), whereas HLA-B*81:01 expressing individuals showed selection of variants at P6 of the epitope (L76V/T/I, L76X, P = 2×10^−43^) with no selection mediated by HLA-B*42:01 and HLA-B*42:02 on this RM9-Nef epitope (Figure 2F) (Table 1).

**Table 1:** HLA-B*07:02, 42:01, 42:02 and 81:01-associated TL9-p24 and RM9-Nef HIV-1 polymorphisms

**Epitope**	**HLA-B**	**HXB2 location**	**Escape variant selected** ^**a**^	**Sequence** ^**b**^	**% HLA match**	**% HLA mismatch**	**P**	**Q** ^**c**^	**N**
TL9-p24	42:01	182	T	EGA*TP* ***T*** *DLNTML*NTV	9	1	7.0E-07	2.4E-04	1279
	81:01	177	D	**D**GA*TPQDLNTML*NTV	7	1	4.4E-05	1.1E-02	1283
	81:01	182	E	EGA*TP* ***E*** *DLNTML*NTV	5	0	2.1E-08	7.6E-06	1282
	81:01	182	G	EGA*TP* ***G*** *DLNTML*NTV	8	1	6.2E-06	1.8E-03	1281
	81:01	182	S	EGA*TP* ***S*** *DLNTML*NTV	21	3	6.2E-12	4.3E-09	1277
	81:01	186	S	EGA*TPQDLN* ***S*** *ML*NTV	31	3	1.8E-24	8.5E-21	1285
	81:01	191	I	EGA*TPQDLNSML*NT**I**	9	2	3.2E-06	9.6E-04	1288
RM9-Nef	07:02	71	K	FPV***K*** *PQVPLRPM*TYK	41	6	7.4E-05	2.4E-02	1293
	42:02	81	F	FPV*RPQVPLRPM*T**F**K	34	9	1.4E-04	4.0E-02	1126
	81:01	76	V	FPV*RPQVP* ***V*** *RPM*TYK	34	6	3.3E-16	4.8E-13	1327
	81:01	76	T	FPV*RPQVP* ***T*** *RPM*TYK	17	1	1.9E-15	2.3E-12	1327
	81:01	76	I	FPV*RPQVP* ***I*** *RPM*TYK	7	0	4.0E-06	2.1E-03	1327

^a^Escape polymorphism shows the amino acid selected in that particular HXB2 location, also indicated by bold face.

^b^Epitope is italics with the consensus shown for ^+^/- 3 amino acids; bold indicates the site of polymorphism.

^c^Only Q-values <0.05 are included.

Thus, HLAI micropolymorphisms among these 4 HLA-B alleles result in distinct patterns of immunodominance at a population level. However, even when the frequency of recognition of epitopes was similar, such as TL9-p24 targeting in HLA-B*42:01-positive or HLA-B*81:01-positive subjects; and RM9-Nef targeting in subjects expressing any of the 4 closely-related B7 superfamily HLAI molecules, selection pressure on HIV-1 was HLAI-specific.

### A conformational switch induces altered presentation of TL9-p24 by HLA-B*81:01 compared to HLA-B*07:02 and HLA-B*42:01

We hypothesised that differential HLAI-specific selection pressure operating on the same epitope may result from structural differences in the HLA-peptide complex. To explore this notion, we first solved the atomic structures of HLA-B*07:02, HLA-B*42:01 and HLA-B*81:01 with TL9-p24. In addition, we solved the atomic structures of HLA-B*07:02, HLA-B*42:01, HLA-B*42:02 and HLA-B*81:01 with RM9-Nef (see below). All structures were determined to extremely high resolutions, between 1.18 Å and 2.09 Å, with crystallographic R_work_/R_free_ ratios within accepted limits as shown in the theoretically expected distribution [19] (Additional file 1: Tables S1 and S2). The electron density around the HLAI binding groove and the peptide was unambiguous in all of the structures.

**Figure 3:**
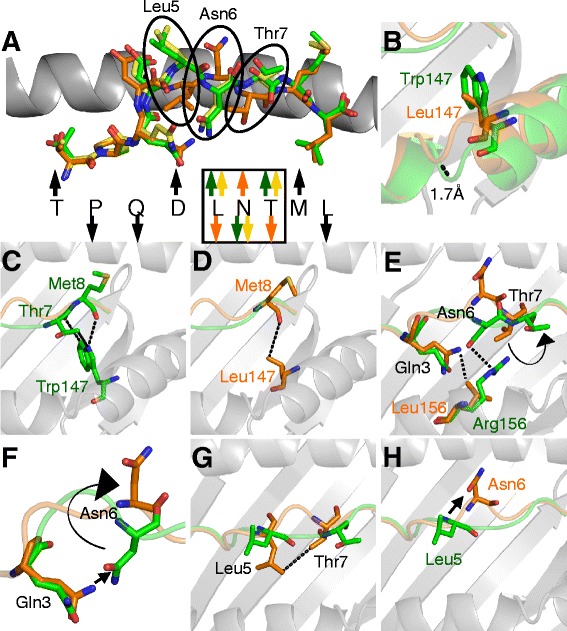
TL9-p24 exhibits a unique conformation when presented by HLA-B*81:01. Comparison of the presentation modes of HLA-B*07:02-TL9 (green sticks), HLA-B*81:01-TL9 (orange sticks) and HLA-B*42:01-TL9 (yellow sticks), the HLAI binding groove is shown as grey cartoon. **(A)** Structural alignment of HLA-B*07:02-TL9, HLA-B*81:01-TL9 and HLA-B*42:01-TL9 peptide conformations showing that HLA-B*07:02 and HLA-B*42:01 present TL9-p24 in identical conformations, whereas in HLA-B*81:01 residues 5–7 in TL9-p24 are presented in a distinct conformation (arrows pointing up indicate that the corresponding residue is solvent exposed and available for TCR contact, arrows pointing down indicate that the corresponding residue is buried in the HLA groove; black arrows designate that the residue is in the same orientation in all three structures, colored arrows indicate different residue conformations according to the colors used in each structure). The positions of the circled residues (Leu5, Asn6 and Thr7) may be important to explain differential escape when presented by different HLAIs. **(B)** HLA-B*07:02 and HLA-B*42:01 contain Trp at residue 147, compared to Leu in HLA-B*81:01. This difference generates a 1.7 Å shift in the HLAα1 helix. **(C, D)** This shift alters interactions between the HLAI binding groove and residues 7 and 8 in the peptide. **(E)** HLA-B*07:02 and HLA-B*42:01 contain Arg at residue 156 compared to Leu156 in HLA-B*81:01. This polymorphism alters interactions with the peptide leading to the observed divergent conformations. **(F)** TL9-p24 residue Gln3 adopts a slightly different conformation in HLA-B*07:02 and HLA-B*42:01 compared to HLA-B*81:01 which contributes to the different position of Asn6 in the peptide. **(G)** TL9-p24 residues Leu5 and Thr7 form a stabilising interaction in the HLA-B*81:01-TL9 structure that is not present in HLA-B*07:02-TL9, or HLA-B*42:01-TL9. **(H)** In HLA-B*07:02-TL9 and HLA-B*42:01-TL9, residue Leu5 is the most solvent exposed residue. However, in HLA-B*81:01-TL9, residue Asn6 assumes this role.

The total number of contacts, buried surface area and surface complementarities were comparable in TL9-p24 structures in complex with HLA-B*07:02, HLA-B*42:01 and HLA-B*81:01 (Table 2). The overall conformation of the TL9-p24 peptide when presented by HLA-B*07:02 and HLA-B*42:01 was virtually identical, with Pro2 and Leu9 acting as the main peptide anchor residues and Asp4, Leu5, Thr7 and Met8 pointing away from the groove for potential interactions with TCRs (Figure 3A). Although peptide positions 1–4 and 8–9 were in similar orientations in both HLA-B*07:02-TL9, HLA-B*42:01-TL9 and HLA-B*81:01-TL9 structures, residues 5–7 in the HLA-B*81:01-TL9 structure, which differs in the peptide-presentation platform by just 4 and 3 residues compared to HLA-B*07:02 and HLA-B*42:01, respectively, were flipped into the opposite orientation (Figure 3A). This ‘conformational switch’ resulted in Leu5 and Thr7 acting as secondary anchor residues in HLA-B*81:01-TL9, as opposed to being solvent exposed in HLA-B*07:02-TL9 and HLA-B*42:01-TL9 complexes. In the HLA-B*81:01-TL9 complex, Asn6 of the peptide acted as the dominant feature pointing out of the groove. In contrast, the same residue served as a secondary anchor residue in HLA-B*07:02-TL9 and HLA-B*42:01-TL9. Thus, unexpected and striking differences in TL9-p24 conformation transform the epitope from the perspective of an incoming TCR restricted by HLA-B*81:01 compared to HLA-B*07:02 and HLA-B*42:01.

**Table 2:** **HLA-TL9 contact table**

	**B0702**		**B8101**		**B4201**	
**TL9**	**VdW**	**HB/SB**	**VdW**	**HB/SB**	**VdW**	**HB/SB**
Thr1	26	4	27	5	23	4
Pro2	24	0	22	0	21	0
Gln3	28	1	26	1	24	2
Asp4	4	1	6	2	5	1
Gln5	0	0	2	1	0	1
Asn6	15	1	6	0	4	1
Thr7	9	3	5	2	7	0
Met8	23	2	8	0	25	2
Leu9	23	4	15	3	24	4
Total	152	16	117	14	133	15
BSA (Å)	1651.8		1726.2		1654.2	
SC (Å)	0.741		0.741		0.714	

HB = hydrogen bond, SB = salt bridge, vdW = van der Waals interactions, BSA = buried surface area, SC = surface complementarity.

*A 3.4 Å cut-off was used for HBs and SBs, and a 4 Å cut-off was used for vdW.

To explain how minor HLAI polymorphisms could account for this distinct peptide conformation, we focused on two polymorphic residues, at position 147 and 156 of the three HLAI molecules presenting TL9-p24. HLA-B*07:02 and HLA-B*42:01 have Trp at position 147, whereas HLA-B*81:01 has Leu147 (Figure 3B). This side chain had a substantial impact on the position of the α2 helix of the HLAI, evident by a movement of 1.7 Å towards the peptide for HLA-B*81:01 (Figure 3B), and altered the interaction between the HLAI and the C-terminus of the TL9-p24 peptide. Trp147, in HLA-B*07:02 and HLA-B*42:01, made three van der Waals (VdW) contacts with Thr7 and Met8 in the peptide, compared to just one VdW contact between Leu147 in HLA-B*81:01 and Met8 (Figure 3C, D), contributing to the respective peptide conformations.

A second important difference occurred at HLAI residue 156 (with Arg, Asp and Leu at this position in HLA-B*07:02, HLA-B*42:01 and HLA-B*81:01 respectively) which contributed to the conformational differences observed at peptide residues Gln3, Asn6 and Thr7 (Figure 3E, showing HLA-B*07:02 and HLA-B*81:01). The altered position of peptide residue Gln3 in HLA-B*81:01, compared to that in HLA-B*07:02 and HLA-B*42:01, forced Asn6 to swing up, out and clear of Gln3 in HLA-B*81:01 (Figure 3F). The smaller side chain of Leu156 in HLA-B*81:01 compared to the longer Arg156 in HLA-B*07:02 and Asp156 in HLA-B*42:01 (Figure 3E), enabled peptide residues Leu5 and Thr7 to form interactions stabilizing Asn6 in the flipped orientation in the HLA-B*81:01 structure (Figure 3G). Overall, therefore, the altered network of contacts between the TL9-p24 peptide and the polymorphic residues in HLA-B*81:01, compared to those of HLA-B*07:02 and HLA-B*42:01, resulted in the upward display of different residues within the solvent exposed, central peptide bulge (Figure 3H).

### HLAI micropolymorphisms alter direct interactions with the RM9-Nef peptide, explaining differences in pHLAI stability and overall epitope presentation

**Figure 4:**
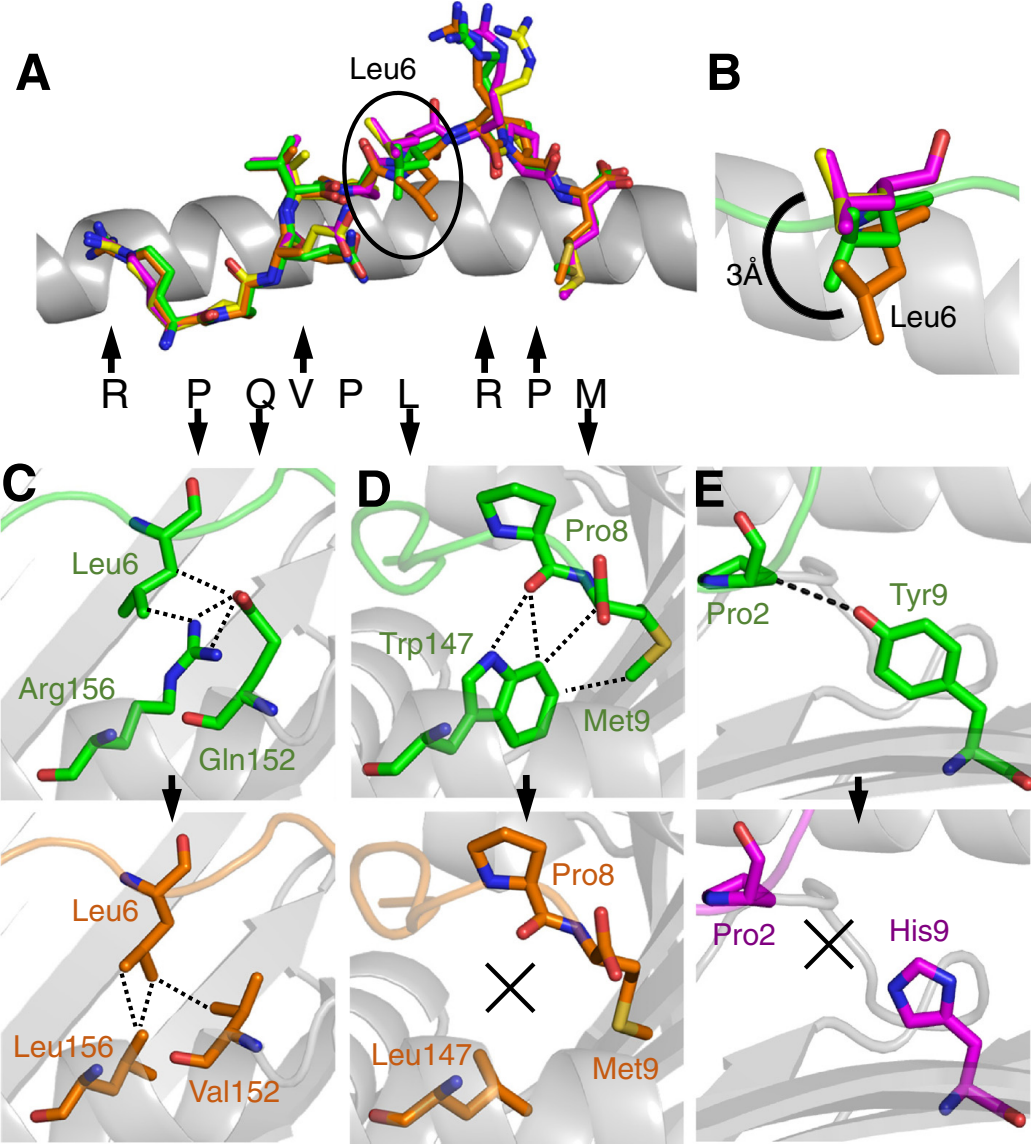
**HLAI polymorphisms contribute to differences in the fine presentation mode of RM9-Nef.** Comparison of the presentation modes of HLA-B*07:02-RM9 (green sticks), HLA-B*81:01-RM9 (orange sticks), HLA-B*42:01-RM9 (yellow sticks) and HLA-B*42:02-RM9 (pink sticks), the HLAI binding groove is shown as grey cartoon. **(A)** Structural alignment of HLA-B*07:02-RM9, HLA-B*81:01-RM9, HLA-B*42:01-RM9 and HLA-B*42:02-RM9 peptide conformations showing differences around peptide residues Arg1, Leu6 and Arg7 (black arrows pointing up indicate that the corresponding residue is solvent exposed and available for TCR contact, black arrows pointing down indicate that the corresponding residue is buried in the HLA groove; no arrow indicates a position between solvent exposed and buried). The position of the circled residue (Leu6) may be important to explain differential escape when presented by different HLAIs. **(B)** RM9-Nef residue Leu6 undergoes a 3 Å shift in position in the HLA-B*81:01 structure compared to HLA-B*42:01 and HLA-B*42:02. **(C)** HLA-B*81:01 contains a Leu at position 156 compared to Arg in HLA-B*07:02 and Asp in HLA-B*42:01 and HLA-B*42:02. This polymorphism alters the interactions between RM9-Nef residue Leu6 and the different HLAIs contributing towards its conformational heterogeneity. **(D)** HLA-B*81:01 contains a Leu at position 147 compared to Trp in HLA-B*07:02, HLA-B*42:01 and HLA-B*42:02. This polymorphism reduces the interactions between residues Pro8 and Met9 in the peptide and HLA-B*81:01 compared to the other HLAIs. **(E)** HLA-B*42:02 contains a His at position 9 compared to Tyr in HLA-B*07:02, HLA-B*81:01 and HLA-B*42:01. This polymorphism reduces the interactions between residue Pro2 in the peptide and HLA-B*42:02 compared to the other HLAIs.

We next analyzed the structures of HLA-B*07:02, HLA-B*42:01, HLA-B*42:02 and HLA-B*81:01 in complex with the well characterised RM9-Nef epitope. Pro2 and Met9 acted as primary anchor residues and Arg1, Val4 and Arg7 pointed away from the groove for potential TCR interactions (Figure 4A). The total number of contacts, buried surface area and surface complementarities were comparable in all four structures (Table 3). Although the overall conformation of the RM9-Nef peptide backbone was similar for all 4 HLAI molecules, we observed important differences in the side chain orientations that could potentially impact T-cell recognition and viral escape, as detailed below.

**Table 3:** **HLA-RM9 contact table**

	**B0702**		**B8101**		**B4201**		**B4202**	
**TL9**	**VdW**	**HB/SB**	**VdW**	**HB/SB**	**VdW**	**HB/SB**	**VdW**	**HB/SB**
Arg1	37	5	35	4	30	4	30	4
Pro2	23	0	22	0	26	0	21	1
Gln3	27	1	23	2	24	2	26	2
Val4	2	0	2	0	2	0	2	0
Pro5	12	0	10	1	8	0	9	0
Leu6	17	0	6	0	4	0	10	0
Arg7	17	4	4	0	11	0	11	0
Pro8	9	1	9	0	15	1	14	1
Leu9	31	5	30	5	56	10	30	4
Total	175	16	141	12	176	17	153	12
BSA (Å)	1813.8		1843.4		1720.2		1796	
SC (Å)	0.757		0.751		0.797		0.807	

HB = hydrogen bond, SB = salt bridge, vdW = van der Waals interactions, BSA = buried surface area, SC = surface complementarity.

*A 3.4 Å cut-off was used for HBs and SBs, and a 4 Å cut-off was used for vdW.

Despite distinct selection pressure at position 1 of RM9-Nef by HLA-B*07:02 (R71K) (Figure 2F) (Table 1), peptide residue Arg1 was in a similar conformation in all 4 HLAIs (Figure 4A). However, the altered conformation of Leu6 in the RM9-Nef-HLA-B*81:01 complex, compared with the other three complexes has similarities with the conformational switch described above in relation to TL9-p24. Leu6 was in an identical position within HLA-B*42:01 and HLA-B*42:02, but moved by 3 Å in HLA-B*81:01 and 1.5 Å in HLA-B*07:02 (Figure 4B). These differences were mediated by polymorphisms at residues 156 and 152 within the α2 helix. The larger side chains of Arg156 and Gln152 in HLA-B*07:02 pushed Leu6 up compared to the smaller side chains of Leu156 and Val152 in HLA-B*81:01 (Figure 4C), changes which may be linked to the L76V/T/I selection that is mediated only by HLA-B*81:01.

The other two major differences in RM9-Nef peptide presentation were located at the junction between HLAI residue 147 and peptide residue Pro8, and between HLAI residue Tyr9 and peptide residue Pro2. The smaller side chain of Leu147 in HLA-B*81:01 formed no contacts with Pro8, whereas Trp147 in the 3 other HLAIs made multiple interactions with Pro8 (Figure 4D). Similarly, in all but HLA-B*42:02, in which the side chain of His9 was too short, Tyr9 could contact Pro2 (Figure 4E). These differences could explain the slightly reduced stability of RM9 bound to HLA-B*81:01 and HLA-B*42:02 (Figure 2B,D).

## Discussion

Taken together, these data demonstrate that, from the perspective of the TCR, TL9-p24 ‘looks’ completely different in the context of HLA-B*81:01 than it does in the context of HLA-B*07:02 or HLA-B*42:01. This difference in potential TCR contact residues is consistent with the differential viral escape patterns observed between these closely related HLAI molecules. Indeed, 4 of these differences are *only* selected by HLA-B*81:01, of which two impact on viral replicative capacity [13].

We were unable to generate enough soluble HLA-B*42:02-TL9 protein to generate a crystal structure, consistent with the low stability and lack of recognition of this pHLAI [7]. HLA-B*42:02 has a unique polymorphism within the B-pocket and contains His at residue 9 as opposed to Tyr9 in HLA-B*07:02, HLA-B*42:01 and HLA-B*81:01 molecules. Tyr9 in HLA-B*07:02, HLA-B*42:01 made direct contacts with the primary N-terminal anchor residue (Pro2) in the TL9-p24 peptide. Thus, His at peptide position 9 could alter this interaction and destabilise the HLA-B*42:02-TL9 complex. This notion is consistent with the lack of detectable responses observed to TL9-p24 in HLA-B*42:02 individuals [7], and is most likely a result of the low HLA-B*42:02-TL9 stability demonstrated here by both peptide-HLAI off-rates and by temperature dependent circular dichroism experiments.

The four closely-related RM9-Nef peptide-HLAI structures show that similar conformations do not preclude HLAI-specific selection pressures, such as for R71K, that is only observed in the context of HLA-B*07:02. Our structural analysis demonstrated that R71 (position 1 in the peptide) pointed up, away from the HLAI groove and could therefore act as a putative TCR contact. Indeed, our previous work has shown that N-terminal peptide residue 1 can serve as an important TCR contact [20] in some systems. However, direct binding by a TCR to this residue would depend on the overall orientation of the TCR as most contacts are usually made with the central bulge of the peptide (normally residues 4–6 in the canonical 9-mer peptide). Thus, sensitivity to changes at peptide N-terminal position 1 would likely be highly dependent on the TCR sequence deployed by T-cells recognising RM9-Nef in the context of divergent HLAIs. Because the selection of self-ligands during thymic education are likely different depending on the HLA type, we speculate that HLA-B*07:02 expressing individuals may select a TCR, sensitive to the R71K mutation, that is not selected in HLA-B*42:01/42:02/81:01 positive individuals. TCR structures obtained from HLA-B*07:02 restricted RM9 specific T-cell clones sensitive to the R71K mutation are needed to investigate this notion further. These findings further highlight that HLA-allele specific HIV sequence changes at a population level are a highly sensitive measure of HLA-allele specific selective immune pressure.

Finally, these data demonstrate that a substantial alteration of the conformation of peptide residue P6 in the HLA-B*81:01-RM9 complex is, as with the TL9-p24 Gag-HLA-B*81:01 structure, associated with the differential selection of escape mutations observed *in vivo*. These observations in relation to both the TL9-p24 and RM9-Nef epitopes support the notion that different restriction elements presenting the same viral epitope in a structurally distinct conformation have an impact on the patterns of viral escape and, thereby, potentially also on immune control.

## Conclusions

These data suggest that identical peptides presented through subtly different HLAI alleles can be recognized as distinct epitopes and provide a novel structural mechanism for previously observed differential HLA allele specific patterns of HIV-1 escape and disease progression.

## Methods

### Study subjects and HIV-1 sequence analysis

We studied 1,327 adults with chronic antiretroviral therapy (ART)-naïve C-clade HIV-1 infection recruited from Durban, South Africa [8,9] and from the Thames Valley Cohort, United Kingdom [21]. Informed consent was obtained from all participating individuals, and institutional review boards at the University of KwaZulu-Natal, Massachusetts General Hospital, and the University of Oxford approved the study. HIV-1 sequences from Gag and Nef proteins were generated [8] and analyzed [9] as previously described.

### IFNγ ELISPOT

The HIV-1 specific CD8^+^ T-cell responses were determined in gamma interferon (IFNγ) enzyme-linked immunospot (ELISPOT) assays. Frequencies of individuals targeting TL9-p24 contained within overlapping peptide (OLP)-25 (GA*TPQDLNTML*NTVGGH) and individuals targeting RM9-Nef contained within OLP-76 (EVGFPV*RPQVPLRPM*TFK) were determined by screening a total of 1,009 individuals as previously described [8,14,22], of which n = 76 where HLA-B*07:02^+^, n = 154 where HLA-B*42:01^+^, n = 22 where HLA-B*42:02^+^ and n = 96 where HLA-B*81:01^+^. Individuals expressing HLA-B*39:10 (n = 30) were excluded as they also target TL9-p24 and RM9-Nef.

### pHLAI stability assays

The measurement of pHLAI stability was determined with a dissociation assay based on radiolabeled β2m and biotinylated HLAI, as recently described [23]. Briefly, biotinylated HLAI heavy chain, ^125^I-labeled β2m, and peptide were allowed to fold into pHLAI complexes in streptavidin-coated scintillation microplates (Flashplate PLUS, Perkin Elmer, Boston, MA) for 24 h at 18°C. Excess of unlabeled β2m was added and dissociation was initiated by placing the microplate in a scintillation reader (TopCount NXT, Perkin Elmer, Boston, MA) operating at 37°C. The scintillation signal was monitored by continuous reading of the microplate for 24 h. Half-lives were calculated from dissociation curves using the exponential decay equation in Prism v.5.0a (GraphPad, San Diego, CA). Assays were performed in duplicate; the mean value of two experiments is reported.

Additionally, the thermal stability of HLA-B complexes was assessed by circular dichroism (CD) spectroscopy monitoring the change in ellipticities at 218 nm. Data were collected on an Aviv Model 215 spectropolarimeter (Aviv Biomedical Inc., Lakewood, NJ) using an 0.1-cm quartz cell. Proteins were dissolved in PBS at concentrations of 3 μM. Melting curves were recorded in 0.5°C intervals from 4°C up to a maximum temperature when protein aggregation was observed. Melting curves were analyzed assuming a two-state trimer-to-monomer transition from the native (N) to unfolded (U) conformation N_3_ ↔ 3U with an equilibrium constant K = [U]^3^/[N_3_] = F/[3c^2^(1-F)^3^] where F and c are the degree of folding and protein concentration, respectively. Data were fitted as described [24]. Fitted parameters were the melting temperature T_m_, van’t Hoff’s enthalpy ΔH_vH_, and the slope and intercept of the native baseline. As all protein complexes aggregated to various degrees upon unfolding, the ellipticity of the unfolded state was set as a constant of −1.36 M^−1^ cm^−1^ [25].

#### Construct design

The HLAI heavy chains and β2m chain were generated by PCR mutagenesis (Stratagene) and PCR cloning. All sequences were confirmed by automated DNA sequencing (Lark Technologies). The HLAI heavy chains (residues 1–248) (α1, α2 and α3 domains), and β2m (residues 1 – 100) were also cloned and used to make the pHLAI complexes. The HLAI α chains and β2m sequences were inserted into separate pGMT7 expression plasmids under the control of the T7 promoter [2].

#### Protein expression, refolding and purification

Competent Rosetta DE3 *E.coli* cells were used to produce the HLAI heavy chains and β2m in the form of inclusion bodies (IBs) using 0.5 mM IPTG to induce expression and proteins were chemically refolded as described previously [26,27].

#### Crystallization, diffraction data collection and model refinement

All protein crystals were grown at 18°C by vapour diffusion via the sitting drop technique. 200 nL of each pHLAI (10 mg/ml) in crystallization buffer (10 mM Tris pH 8.1 and 10 mM NaCl) was added to 200 nL of reservoir solution. HLA-B*07:02-TL9 crystals were grown in 22% PEG 4000 and 0.2 M ammonium sulphate, 0.1 M sodium acetate [28]; HLA-B*81:01-TL9 crystals were grown in TOPS4 in 0.1 M HEPES pH 7.0, 20% PEG 4000 and 0.2 M ammonium sulphate [28]; HLA-B*42:01-TL9 crystals were grown in 10% PEG 6000, 10 mM magnesium chloride; HLA-B*07:02-RM9 crystals were grown in TOPS in 0.1 M sodium cacodylate pH 6, 15% PEG 8000 and 15% glycerol [28]; HLA-B*81:01-RM9 crystals were grown in TOPS in 0.1 M Tris pH 8.0, 15% PEG 4000 and 15% glycerol [28]; HLA-B*42:01-RM9 crystals were grown in 10% PEG 6000, 10 mM magnesium chloride; HLA-B*42:02-RM9 crystals were grown in 10% PEG 6000, 10 mM magnesium chloride [28]. All crystals were soaked in 30% ethylene glycol before cryo-cooling. All crystallization screens and optimization experiments were completed using an Art-Robbins Phoenix dispensing robot (Alpha Biotech Ltd, UK). Data were collected at 100 K at the Diamond Light Source, Oxfordshire, UK. All datasets were collected at a wavelength of 0.98 Å using an ADSC Q315 CCD detector. Reflection intensities were estimated with the XIA2 package (Winter G.) and the data were scaled, reduced and analyzed with SCALA and the CCP4 package (Collaborative Computational Project 1994). Structures were solved with molecular replacement using PHASER [29]. Sequences were adjusted with COOT [30] and the models refined with REFMAC5. Graphical representations were prepared with PYMOL (DeLano, 2002). The reflection data and final model coordinates were deposited with the PDB database (HLA-B*07:02-TL9, PDB: 4U1H; HLA-B*81:01-TL9, PDB: 4U1I; HLA-B*42:01-TL9, PDB: 4U1J; HLA-B*07:02-RM9, PDB: 4U1K; HLA-B*81:01-RM9, PDB: 4U1L; HLA-B*42:01-RM9, PDB: 4U1M; HLA-B*42:02-RM9, PDB: 4U1N).

## Additional file

Additional file 1: Tables S1 and S2. Supplementary information.

## Abbreviations

HLAI: Human leukocyte antigen class I
TCR: T cell receptor
