# Supplementary material for: A molecular switch in immunodominant HIV-1-specific CD8 T-cell epitopes shapes differential HLA-restricted escape
Source: Retrovirology. 2015 Feb 20;12:20. doi: 10.1186/s12977-015-0149-5 (PMC4347545; doi:10.1186/s12977-015-0149-5)
Supplement: Additional file 1: Tables S1 and S2. — Supplementary information. [file 12977_2015_149_MOESM1_ESM.docx]

**Supplementary information:**

**A MOLECULAR SWITCH IN IMMUNODOMINANT HIV-1-SPECIFIC CD8 T-CELL GAG EPITOPES SHAPES DIFFERENTIAL HLA-RESTRICTED ESCAPE**

Henrik N. Kløverpris^1,5*^, David K. Cole^2*^, Anna Fuller^2*^, Jonathan Carlson^3^, Konrad Beck^4^, Andrea A.J. Schauenburg^2^, Pierre J. Rizkallah^2^, Søren Buus^5^, Andrew K. Sewell^2*^, and Philip Goulder^6*^

*^1^KwaZulu- Natal Research Institute for Tuberculosis and HIV, K-RITH, Nelson R Mandela School of Medicine, University of KwaZulu-Natal, South Africa.*

*^2^Cardiff University School of Medicine, Heath Park, Cardiff, UK.*

*^3^Microsoft Research, eScience Group, Los Angeles, CA 90024, USA*

*^4^Cardiff University School of Dentistry, Heath Park, Cardiff, UK.*

*^5^Department of International Health, Immunology and Microbiology, University of Copenhagen, 2200-Copenhagen N, Denmark.*

*^6^Department of Paediatrics, University of Oxford, Peter Medawar Building, OX1 3SY, UK*

**^*^**These authors contributed equally to this study.

Correspondence:

Dr Henrik N. Kløverpris, K-RITH: KwaZulu-Natal Research, Institute for Tuberculosis and HIV, Durban, South Africa, henrik.kloverpris@k-rith.org

or Dr David Cole, Cardiff University School of Medicine, UK, E-mail: coledk@cf.ac.uk.

**Supplementary Tables:**

**Supplementary Table S1.** Data collection and refinement statistics for HLA-TL9 structures

|  | B0702-TL9 | B8101-TL9 | B4201-TL9 |
| --- | --- | --- | --- |
| PDB | 4U1H | 4U1I | 4U1J |
| **Data collection** |  |  |  |
| Space group | P21 21 21 | P21 21 21 | P21 21 21 |
| **Cell dimensions** |  |  |  |
| *a*, *b*, *c* (Å) | 51.0, 81.4, 110.1 | 51.3, 81.5, 110.1 | 50.9, 81.7, 111.2 |
| α, β, γ (°) | 90, 90, 90 | 90, 90, 90 | 90, 90, 90 |
| Resolution (Å) | 55-1.59 | 45.6-1.92 | 55.6-1.38 |
| *R*_merge_ (%) | 7.3 | 23.1 | 10.7 |
| *I* / σ*I* | 16.5 | 11.8 | 9.9 |
| Completeness (%) | 99.9 | 99.7 | 100 |
| Redundancy | 7.3 | 7.4 | 7.2 |
| **Refinement** |  |  |  |
| Resolution (Å) | 1.59 | 1.92 | 1.38 |
| No. reflections | 59,167 | 34,045 | 90,415 |
| No reflections in Rfree set | 3,157 | 1,793 | 4,773 |
| *R*_work_ / *R*_free_ (%) | 16.4/19.4 | 17.7/20.8 | 17/19.6 |
| **R.m.s. deviations** |  |  |  |
| Bond lengths (Å) | 0.019 | 0.019 | 0.019 |
| Bond Angles (°) | 1.944 | 1.945 | 1.939 |
| Mean B value (Å^2^) | 23.7 | 26.2 | 19.6 |
| Overall coordinate error (Å) | 0.049 | 0.094 | 0.038 |

* One crystal was used for solving each structure.

**Supplementary Table S2.** Data collection and refinement statistics for HLA-RM9 structures

|  | B0702-RM9 | B8101-RM9 | B4201-RM9 | B4202-RM9 |
| --- | --- | --- | --- | --- |
| PDB | 4U1K | 4U1L | 4U1M | 4U1N |
| **Data collection** |  |  |  |  |
| Space group | P1 | P1 | P21 21 21 | P21 21 21 |
| **Cell dimensions** |  |  |  |  |
| *a*, *b*, *c* (Å) | 46.3, 49.1, 107.5 | 46.0, 49.1, 107.8 | 51.1, 81.3, 110.9 | 51.0, 81.2, 110.5 |
| α, β, γ (°) | 90, 90, 90 | 91, 93.5, 95.7 | 90, 90, 90 | 90, 90, 90 |
| Resolution (Å) | 49-2.09 | 53.8-2.06 | 55.5-1.18 | 81.2-1.77 |
| *R*_merge_ (%) | 9.8 | 10.1 | 5.3 | 9.7 |
| *I* / σ*I* | 4.8 | 5.8 | 17.5 | 17.7 |
| Completeness (%) | 97.5 | 97.7 | 99.1 | 100 |
| Redundancy | 2.0 | 2.0 | 7.1 | 7.3 |
| **Refinement** |  |  |  |  |
| Resolution (Å) | 2.09 | 2.06 | 1.18 | 1.77 |
| No. reflections | 51,450 | 53,719 | 143,022 | 43,193 |
| No reflections in Rfree set | 2,757 | 2,874 | 7,561 | 2,299 |
| *R*_work_ / *R*_free_ (%) | 17.5/22.6 | 17.2/20.9 | 16.2/18 | 16.2/19.9 |
| **R.m.s. deviations** |  |  |  |  |
| Bond lengths (Å) | 0.017 | 0.019 | 0.024 | 0.019 |
| Bond Angles (°) | 1.857 | 1.949 | 2.438 | 1.943 |
| Mean B value (Å^2^) | 51.5 | 48.7 | 18 | 22.5 |
| Overall coordinate error (Å) | 0.145 | 0.133 | 0.024 | 0.068 |

* One crystal was used for solving each structure.
